# Barriers and facilitators to changing bowel care practices after spinal cord injury: a Theoretical Domains Framework approach

**DOI:** 10.1038/s41393-021-00743-0

**Published:** 2022-01-07

**Authors:** Vera-Ellen M. Lucci, Rhyann C. McKay, Christopher B. McBride, Maureen S. McGrath, Rhonda Willms, Heather L. Gainforth, Victoria E. Claydon

**Affiliations:** 1 Department of Biomedical Physiology and Kinesiology, Simon Fraser University, Burnaby, BC Canada; 2 International Collaboration on Repair and Discoveries (ICORD), University of British Columbia, Vancouver, BC Canada; 3 School of Health and Exercise Sciences, University of British Columbia Okanagan, Kelowna, BC Canada; 4 Spinal Cord Injury British Columbia, Vancouver, BC Canada; 5 GF Strong Rehabilitation Centre, Spinal Cord Injury Program, Vancouver Coastal Health, Vancouver, BC Canada; 6 Division of Physical Medicine and Rehabilitation, Faculty of Medicine, UBC, Vancouver, BC Canada

**Keywords:** Quality of life, Neurophysiology, Translational research

## Abstract

**Background:**

Improvement to autonomic processes such as bladder, bowel and sexual function are prioritised by individuals with spinal cord injury (SCI). Bowel care is associated with high levels of dissatisfaction and decreased quality of life. Despite dissatisfaction, 71% of individuals have not changed their bowel care routine for at least 5 years, highlighting a disconnect between dissatisfaction with bowel care and changing routines to optimise bowel care.

**Objective:**

Using an integrated knowledge translation approach, we aimed to explore the barriers and facilitators to making changes to bowel care in individuals with SCI.

**Methods:**

Our approach was guided by the Behaviour Change Wheel and used the Theoretical Domains Framework (TDF). Semi-structured interviews were conducted with individuals with SCI (*n* = 13, mean age 48.6 ± 13.1 years) and transcribed verbatim (duration 31.9 ± 7.1 min). Barriers and facilitators were extracted, deductively coded using TDF domains and inductively analysed for themes within domains.

**Results:**

Changing bowel care after SCI was heavily influenced by four TDF domains: environmental context and resources (workplace flexibility, opportunity or circumstance, and access to resources); beliefs about consequences; social influences (perceived support and peer mentorship); and knowledge (knowledge of physiological processes and bowel care options). All intervention functions and policy categories were considered viable intervention options, with human (61%) and digital (33%) platforms preferred.

**Conclusions:**

Modifying bowel care is a multi-factorial behaviour. These findings will support the systematic development and implementation of future interventions to both enable individuals with SCI to change their bowel care and to facilitate the optimisation of bowel care approaches.

## Introduction

Over 2.5 million individuals are living with the devastating consequences of spinal cord injury (SCI) [1]. In addition to the loss of movement and sensation, SCI is also associated with impaired autonomic control, including cardiovascular dysregulation, and bladder, bowel and sexual dysfunctions [2]. Bowel care problems after SCI are multi-factorial but predominantly relate to neurogenic bowel dysfunction resulting from a lack of central nervous system control [3]. Problems with bowel control stem from disruption to spinal sympathetic pathways, as well as sacral parasympathetic and motor pathways [4]. Accordingly, injured individuals often experience impairments in quality of life related to faecal incontinence, faecal urgency, constipation, haemorrhoids and abdominal distention [3, 5, 6]. In addition to physiological impairments, bowel care after SCI also presents a variety of complex cognitive, affective, social and environmental barriers [5–7].

Our recent survey of 287 individuals with SCI revealed that people with SCI identified bowel care as a key modifiable factor for improving their quality of life [5]. Bowel management was a problem for 78% of respondents: it interfered with personal relationships (60%), and prevented staying (62%), and working (41%), away from home. Bowel management was rated as one of the worst effects of living with SCI. Despite these bowel care concerns, most (71%) respondents had not made any changes to their bowel routine for at least 5 years.

In order to systematically develop evidence-based interventions to support people with SCI to optimise their bowel care, it is imperative to elucidate the barriers and facilitators to changing care. To achieve this aim, we used the Theoretical Domains Framework (TDF) [8], an integrative framework that uses theoretical approaches to design interventions aimed at behaviour change [8–13] that has been applied previously in SCI populations [12, 14]. The TDF is an expansion of the capability, opportunity, motivation–behaviour (COM-B) model [10], that can be linked to intervention functions, policy categories and intervention options using the behaviour change wheel (BCW) [10, 15].

We aimed to use the TDF and BCW to identify the barriers and facilitators to making changes to optimise bowel care for individuals with SCI, permitting the generation of a framework for change for affected individuals. In addition to facilitating the systematic co-development of recommendations for interventions, this process will also identify preferred formats for disseminating bowel care guidelines and interventions among our target audience.

## Methods

This study was approved by the Department of Research Ethics at Simon Fraser University and conforms to the principles outlined in the Declaration of Helsinki [16]. All participants provided written informed consent at the time of screening and verbal informed consent at the time of the interview.

### Design

#### Integrated knowledge translation

Consistent with the guiding principles of integrated knowledge translation [17, 18] and based on the geographical location of the study, Spinal Cord Injury British Columbia (SCI BC) and local SCI clinicians were identified as research users and engaged as partners throughout the research process. The integrated knowledge translation process and partner descriptions are provided in Supplementary Table 1.

### Procedures

#### Recruitment and participants

Members of SCI BC who had agreed to receive information about upcoming research studies were emailed by an SCI BC staff member with study information, eligibility criteria and a link to complete a short online screening survey. This study was also promoted on the research participation section of the International Collaboration on Repair Discoveries (ICORD) website (www.icord.org).

**Fig. 1:**
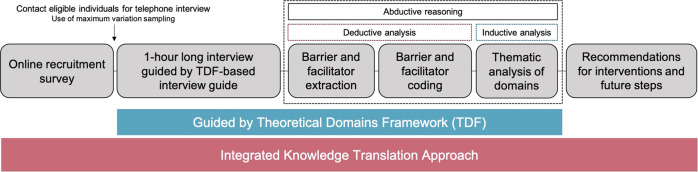
Study protocol. The study incorporated an integrated knowledge translation approach guided by the theoretical domains framework (TDF).

Once invited by email, participants were able to access an online questionnaire and review the consent form. Those who agreed to participate completed a short survey to determine eligibility and provide contact information. Individuals were eligible to participate if they were able to effectively communicate in English, were at least 18 years old, and were living with an SCI in British Columbia, Canada. We employed maximum variation sampling [19] to ensure representation across the SCI community within British Columbia [20], assessing for the following parameters: age, gender, level and completeness of injury, and place of residence (i.e., urban vs. rural). Participants were selected for the interview ensuring representation from the above parameters, in line with the population demographics of British Columbia [20]. Those who completed the survey and met the maximum variation sampling and eligibility criteria were contacted by telephone and a convenient date and time for the interview were scheduled. A schematic of the study protocol can be found in Fig. 1.

#### Interviews

The initial semi-structured interview guide was informed by TDF interview guidance [8]. This interview guide underwent revision by all team members. After pilot testing with a staff member of SCI BC, the interview guide was further refined. Interview guide questions explored barriers to changing bowel care and included probing questions for each domain. Participants were also asked to describe their ideal bowel care intervention including preferred mode of delivery for future interventions.

All interviews were conducted over the phone by the same researcher (V-EML). Interviews were recorded, transcribed verbatim, and anonymised (NVivo, Version 12). Recommendations for using the TDF for interviews include conducting at least ten interviews, with an additional three interviews that were then appraised for the presence of new themes. If new themes emerged, three additional interviews are conducted until no new themes immerge (for a minimum of 13 interviews) [8].

### Data analysis

To fully understand the behaviour, a two-phase abductive data analysis approach was taken, wherein barriers and facilitators to changing bowel care were extracted and analysed both deductively using the TDF, and inductively for themes within each TDF domain.

#### Barrier and facilitator extraction and deductive analyses

Changing bowel care was defined as any action taken to change either bowel routines (acts taken to empty bowels) or bowel programmes (lifestyle actions to optimise bowel emptying). Factors that promoted changing bowel care were coded as facilitators, while factors impeding changing bowel care were coded as barriers. Barrier and facilitator extraction, and subsequent coding into the 14 TDF domains, were performed independently for all interviews by two members of the research team (V-EML, RCM) in duplicate. Agreement between coders was determined using Cohen’s Kappa [21] and prevalence-adjusted bias-adjusted Kappa (PABAK) [22]. Values between 0.61 and 0.80 were considered substantial; values in excess of 0.8 were considered almost perfect [21, 22]. Any disagreement between coders was resolved through discussion. In the event that consensus was not met, an expert coder was consulted (HLG).

#### Inductive analysis

To gain a deeper understanding of the barriers and facilitators identified in the deductive analysis, inductive thematic analysis was conducted within the prevalent TDF domains, whereby a theme is defined as something that ‘captures something important about the data in relation to the research question and represents some level of patterned response or meaning within the data set’ [23]. The inductive analysis was strengthened by the involvement of the research team as ‘critical friends’, where members of the research team (RCM, HLG, VEC) reviewed themes and provided input and suggestions for their refinement [24, 25]. Each critical friend provided their own unique expertise to the research question over a series of three team meetings.

### Intervention and implementation options

#### Intervention options

We identified sources of behaviour using the BCW [10] and linked prominent TDF domains to their associated COM-B components. Using established matrices, COM-B sources of behaviour were mapped to intervention functions, and then to the policy categories most likely to support the intervention function [10].

#### Modes of delivery options

Preferred modes of delivery identified by participants were extracted and independently coded by each coder (V-EML, RCM) using the Modes of Delivery Taxonomy version 0 [26, 27] for all interviews.

## Results

### Participant demographics

Thirteen (*n* = 13) semi-structured interviews were conducted. Participant demographics can be found in Table 1. Participants had a diverse range of SCI levels (C4–L1) and severities (AIS A–D), with interview duration averaging 31.9 ± 7.1 min. The mean age at the time of the interview was 48.6 ± 13.1 years and the average duration since injury was 21.6 ± 12.5 years. The majority of participants were male (*n* = 8) and lived in urban settings (*n* = 8); 12 participants identified as white, with two who also identified as racialised, and one participant who did not report additional demographic information.

**Table 1:** Participant demographics.

Demographic and injury information		
Sample size (F/M)	13 (8/5)	
Age	48.6 ± 13.1 years	
Duration of injury	21.6 ± 12.5 years	
Injury level	C4–L1	
Injury severity	AIS A–D	
Duration of interview	31.9 ± 7.1 min	
Geographical region (urban/rural)	8/5	
Current bowel care strategies used, *N* (%) (multiple responses possible)	Digital stimulus	5 (38%)
	Manual evacuation	1 (8%)
	Suppositories	8 (62%)
	Oral laxatives	6 (46%)
Assistance required, *N* (%)	Total assistance	1 (8%)
	Partial assistance	4 (31%)
	Completely independent	8 (62%)
Position, *N* (%)	Bed	0 (0%)
	Commode	4 (31%)
	Toilet seat	9 (69%)

Where applicable, data are expressed as mean ± SD.

*AIS* American Spinal Injury Association impairment scale.

### Deductive analysis

The two independent coders double-extracted 409 barriers/facilitators from all 13 interviews, comprising 200 barriers and 209 facilitators. All barriers and facilitators were coded into at least one TDF domain, with some items coded into multiple TDF domains, culminating in 426 observations (205 barriers, 221 facilitators). For all barriers and facilitators coded, the average inter-coder Kappa agreement was substantial and PABAK was almost perfect (Cohen’s Kappa 0.76 ± 0.04, PABAK 0.94 ± 0.01). Numbers and proportions of barriers and facilitators identified across all 14 TDF domains can be found in Table 2.

**Table 2:** Barriers and facilitators to changing bowel care by TDF domain.

TDF domain	Barriers			Facilitators			Overall		
	%	*N* occurrences	*N* interviews	%	*N* occurrences	*N* interviews	%	*N* occurrences	*N* interviews
Environmental context and resources	22	46	12	24	54	11	23	100	13
Beliefs about consequences	26	54	13	15	34	12	21	88	13
Social influence	7	14	5	15	34	13	11	48	13
Goals	0	0	0	19	41	13	10	41	13
Reinforcement	12	25	10	7	15	8	9	40	11
Intentions	9	18	7	9	19	9	9	37	11
Knowledge	11	22	8	1	3	3	6	25	8
Memory, attention, and decisions processes	5	10	3	1	3	4	3	13	7
Optimism	5	8	4	2	5	2	3	13	5
Beliefs about capabilities	1	1	1	3	7	5	2	8	6
Emotions	3	7	4	0	0	0	2	7	4
Behavioural regulation	0	0	0	1.5	3	3	0.5	3	3
Skills	0	0	0	1.5	3	2	0.5	3	2
Social/professional roles and identity	0	0	0	0	0	0	0	0	0

*TDF* Theoretical Domains Framework.

**Fig. 2:**
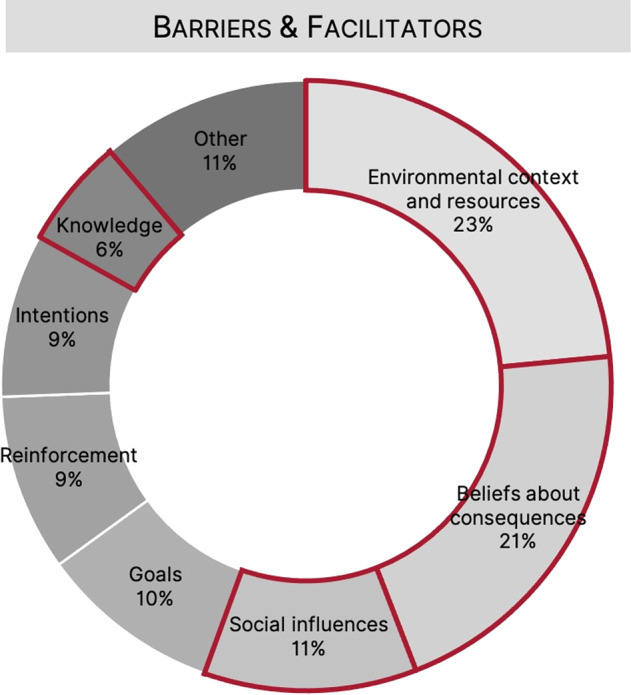
Proportion of total barriers and facilitators by TDF domain across all interviews. Domains outlined in red reflect those included in the inductive analysis. TDF Theoretical Domains Framework.

**Fig. 3:**
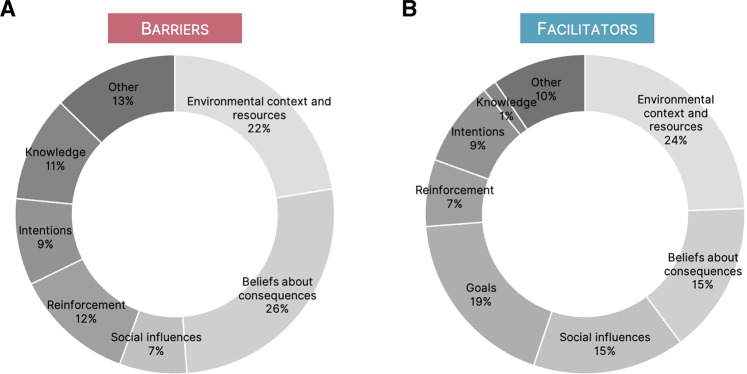
Barriers and facilitators by TDF domain across all interviews. **A** Proportion of barriers by TDF domains. **B** Proportion of facilitators by TDF domains. TDF Theoretical Domains Framework.

Among barriers, the most commonly coded domain was beliefs about consequences (BCon), followed by environmental context and resources (ECR). Together these domains accounted for 48% (*n* = 100) of all reported barriers. Other commonly coded domains for barriers included reinforcement (12%, *n* = 25), knowledge (11%, *n* = 22) and intentions (9%, *n* = 18). Among facilitators, the most commonly coded domain was ECR (24%, *n* = 54), followed by goals (19%, *n* = 41). Across both barriers and facilitators, ECR, BCon and social influences accounted for 55% of all coded barriers and facilitators (ECR, 23%, *n* = 100; BCon, 21%, *n* = 88; social influences, 11%, *n* = 48). A visual representation of all coded barriers and facilitators can be found in Fig. 2 and a representation of TDF domains by either barriers or facilitators can be found in Fig. 3.

### Inductive analysis

**Fig. 4:**
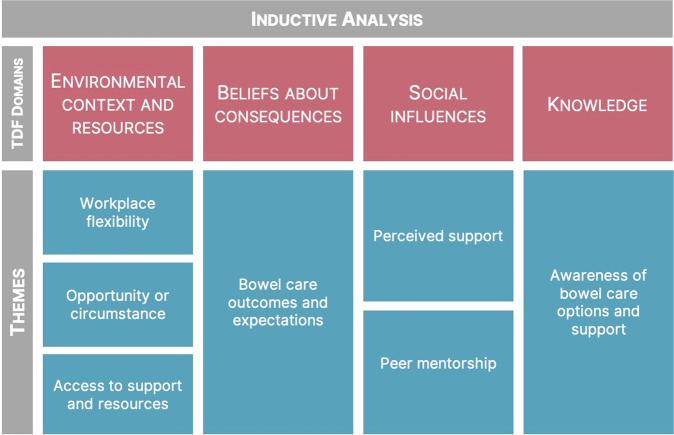
TDF domains and associated themes related to changing bowel care practices after SCI. Red boxes represent TDF domains and blue boxes below represent domain-specific themes. TDF Theoretical Domains Framework.

A thematic analysis was conducted within the three most prominent domains (i.e., ECR, BCon and social influences) and knowledge. These four domains were considered for inductive analysis based on the severity of their implications and their significance in interviews, as determined by the first author. Three members of the research team acted as critical friends (RCM, HLG, VEC), aiding the primary researcher in the selection of prominent domains and refinement of themes. Below, each domain is presented and described, including a description of themes within each domain, and implications for care. Dominant domains and associated themes can be found in Fig. 4.

#### Environmental context and resources

ECR describes any circumstance of a person’s situation or environment that discourages or encourages the development of skill and ability, independence, social competence and adaptive behaviour aimed at changing bowel care. In the interviews, ECR accounted for 23% (*n* = 100) of all coded barriers and facilitators (22% barrier, 24% facilitators). ECR was the most prominent domain and was discussed in every interview. Participants described how flexibility in their workplace influences their decisions to change their bowel care. In addition, one’s opportunity to change and access to resources were also influential in the context of changing bowel care.

##### Workplace flexibility

The theme of workplace flexibility was identified as a facilitator within the ECR domain. When participants were engaged in a supportive and/or flexible work environment, they felt facilitated to make changes to their bowel care routines when desired or necessary. One participant described how the flexibility to work from home allowed them to make changes to their bowel care routine that they may not otherwise have considered:

*“…10 years ago, I started working… out of my home instead of my office, so it’s just easier for me to jump on the toilet if I have to, you know what I mean”*

##### Opportunity or circumstance

In addition to workplace flexibility, one’s individual opportunity or circumstance was shown to influence how individuals approach changes to bowel care. This is particularly important given the reality that approaches to bowel care must change over time to align with physiological changes in bowel function associated with ageing or in response to other lifestyle or physiologic factors. Recognition of circumstances related to ageing was facilitator to changing bowel care. Conversely, some circumstances discussed included tolerance of a suboptimal bowel care routine, where change would only be considered if the routine worsened. Other contextual considerations to changing bowel care included learning about the impact of SCI on individual physiological processes and how best to modify bowel care to the needs of the individual. One participant described it as:

*“It took so long [to empty my bowels] that it was a nightmare and I thought that that was going to be my life. And I [eventually] realized that I needed to [learn] how [my body] responded to laxatives”*

Opportunities to change bowel care sometimes came from changes in other care routines (notably, changes in bladder care) that permitted participants to focus on prioritising changes to bowel care. There was a clear hierarchy in terms of changing care routines, with bladder management conveyed as the less cumbersome routine to change, with more known options and advice from health care professionals:

*“I think I’m in a good spot in terms of if I wanted to change because, like, I found something that really, really helps me manage my bladder”.*

Throughout the interviews, we did not identify any sex differences in bowel care practices with the exception of bladder care. Bladder care was an important consideration for 60% (*n* = 3) of female participants when considering changes to bowel care, which speaks to the interplay between bladder and bowel care.

##### Access to support and resources

Another theme within the ECR domain was access to support and resources. Support (either physical or emotional) was discussed in relation to family members, caregivers and health care professionals, community and SCI peers. A lack of perceived support was often cited as a barrier to change, especially when discussed in the context of physical supports. Lack of support (i.e., ideas for bowel care optimisation, changes to bowel care strategies) from health care professionals decreased participants’ belief in proficiency among health care professionals in relation to bowel care needs and can be seen through the following quotes:

*“I haven’t met anybody that’s been able to offer… information about topics that I bring up with them”*

*“And the thing is that the family doctor, they don’t know much about spinal cord injuries, or neurogenic bladder and bowel. They really only know when something is a disaster, that it needs to be fixed. They’re not like thinking about prevention, or optimizing, you know”*

*“Um… unfortunately, locally, um… I certainly know a lot more than a physiatrist or a doctor, GP… I’m not saying that we’re the doctors, but I know more about bowel than my doctors do”.*

In addition, access to specialists was also discussed geographically, with individuals in rural settings perceiving access to specialist support to be lacking. Physical barriers to changing bowel care included both financial and time restraints. Costs associated with changing bowel care routines but not covered by extended health care were repeated barriers to exploring different bowel care practices. Examples of the impact of both financial and time constraints can be seen through the following quotes:

*“It would depend on the cost totally…”*

*“It’s time consuming and I was busy, still am busy, but it was more for convenient [for me to do my bowel care this way]”*

*“I mean I would have to have some time off work, or I’d have to make a change over the holidays time or something I suppose, but then it would mess up my holidays”.*

#### Beliefs about consequences

BCon accounted for 21% (*n* = 88) of all coded barriers and facilitators (26% barrier, 15% facilitators) and relates to the perceived truth, reality or validity of outcomes of behaviour in a given situation.

##### Bowel care outcomes and expectations

The predominant theme within the BCon domain surrounds bowel care outcomes and expectations, including both participants’ beliefs about changing bowel care (what change can or what cannot do for the participant), and the impact that changing bowel care has on several aspects of life. Fear and apprehension about changing bowel care were widely discussed. One participant described it as:

*“I think fear of accidents would be the biggest… yeah, the biggest challenge for anyone who’s considering changes”.*

In addition, participants often discussed aspects of care that could change as a consequence of changing bowel care, including concerns about losing independence in care routines, with increased dependence on care-aids or family care providers, as highlighted in one interview:

*“I’m independent in every other way, so to not be able to do [an aspect of bowel care] would be… eh… it would be an inconvenience to have someone come in to do that or help with that”*

This focus on bowel care outcomes and expectations was a consistent theme throughout transcripts—all participants discussed BCon, with all participants describing it as a barrier and 12 participants discussing it in terms of facilitating changes to bowel care.

#### Social influences

The social influences domain accounted for 11% (*n* = 48) of all coded barriers and facilitators (7% of all barriers and 15% of all facilitators), highlighting those interpersonal processes that can influence individuals to change their thoughts, feelings, or behaviours. This might include the level of perceived support from interpersonal relationships, perhaps from peer, family or friendship groups, as well as from health care professionals. All interviews discussed social influences as a facilitator, with five interviews also discussing social influences as a barrier to changing bowel care.

##### Perceived support

Participants often regarded the level of perceived support from interpersonal relationships as a barrier or facilitator to changing bowel care. Others’ perceptions, reactions and judgement of bowel care acted as persuasion to change bowel care practices. For example, one participant noted the implication that travelling with friends has on bowel care options and considerations, highlighting that if a bowel care change was perceived to negatively impact interpersonal relationships, it would be a barrier to adoption of the intervention:

*“I went [away] with some friends…and I didn’t want them wiping my bum, you know, one thing to help a buddy get into a shower chair naked or with a towel over my lap, and another thing altogether to stick a finger in [my] bum and wipe [my] ass”.*

In addition to social influences in the context of peer or friendship groups, participants also identified the level of support they receive from health care professionals as influencing making changes to their bowel care. These responses varied, with some participants identifying health care professionals as essential components of bowel care change, whereas others did not regard health care professionals as helpful when exploring this behaviour. These two competing ideas are outlined by the following:

*“My doctor has not been very helpful [in regard to changing bowel care]”*

In contrast to another participant outlining the deep reverence they have for their health care provider when considering changes to bowel care routines and underscoring the complex relationships surrounding making changes to bowel care:

*“I think [changing my bowel care from that my doctor prescribed] would be highly disrespectful and maybe even irresponsible, because, you know it may end up going against you. You know, it may not work out to not take the advice that I’ve been given when I’m lucky enough to have access to that kind of care”.*

Another source of perceived support came from family members, regardless of whether family members were also family care providers. Some individuals spoke about how they felt supported to change their bowel care:

*“I mean, my family would support me [changing my bowel care], obviously”*

While others expressed how they did not feel supported by their family, which was a barrier to changing bowel care practices:

*“But [my partner] did not want to know anything about number two and me, ever”*

##### Peer mentorship

In addition to perceived support, support from peers living with SCI was regarded as highly influential to making bowel care changes. Peer mentorship has repeatedly been shown to have a unique influence on individuals after SCI [28, 29]. Peer mentorship provided relatability regarding bowel care practices while concurrently modelling different approaches to care. It was often mentioned that, when gathered either formally or informally, conversations among individuals with SCI and their peers ultimately turn to the topic of bowel care, giving rise to a common group identity. This empathy, understanding and collectivism was described as:

*“I had been talking to peers at the time. Yeah, and so I made the changes via information from peers”*

*“I have all the peer groups… just relying on the experience of some of the older peers that… might have gone through stuff… every peer coffee at some point has had a discussion on bowels”.*

#### Knowledge

Knowledge accounted for 6% of all coded barriers and facilitators (*n* = 25) and was coded in eight interviews as a barrier, as well as being discussed in three interviews as a facilitator. The principles of thematic analysis emphasise the significance of a theme over the prevalence [23]. Knowledge is the one domain that if unaddressed poses a larger barrier to engaging in other issues surrounding changing bowel care—if one is not aware of the available options, they do not know how to change, and therefore cannot change. The singular theme within the knowledge domain was awareness surrounding bowel care options, implementing changes to bowel care, and support. Lack of awareness included not only the level of understanding of the physiological disruptions to bowel function resulting from SCI, but also knowledge of bowel care options and resources to access those options:

*“I can’t say that because I’m sure somebody has one somewhere, I just don’t know what it is yet” [CONTEXT: do you believe that there is an ideal working routine?]*

*“I didn’t understand anything. I didn’t know why my bladder was not working. I didn’t know why my bowels weren’t working”.*

Conversely, awareness of bowel care options empowered changes to bowel care:

*“I feel like I have the tools and the knowledge I need to change it up if circumstances require it”*

### Identifying intervention options

The TDF domains considered for inductive analysis (BCon, ECR, social influences, knowledge) correspond to the behavioural sources of reflective motivation, both physical and social opportunity, and psychological capability of the COM-B model. After linking COM-B components to intervention functions, all intervention functions could be used to promote bowel care behaviour change. However, enablement, education and training were the three most prominently linked intervention functions. Further linking revealed that any policy category could be considered relevant when developing interventions.

### Identifying implementation options

#### Mode of delivery analysis

Two independent coders (V-EML, RCM) double-extracted 18 modes of delivery from all 13 transcripts. Using the Mode of Delivery Taxonomy version 0 (MoDtv0) [26, 27], extracted modes of delivery were coded into the mode of delivery categories. Inter-coder agreement of mode of delivery coding was almost perfect (Kappa = 0.85 ± 0.2; PABAK = 0.90 ± 0.05). Human (61%), digital platforms (33%) and print material (6%) were identified as potential modes of delivery. It was unclear how human interaction was to be used as a mode of delivery with 82% (*n* = 9) of human modes of delivery coded as ‘unclear’. However, digital and print material included email (15%), websites (23%), instant messages (8%) and leaflets (8%).

## Discussion

These findings provide a theoretical understanding of the barriers and facilitators to changing bowel care practices after SCI. Our analyses reveal that interventions focused on bowel care change should target reflective motivation, psychological capability and both social and physical opportunity. As such, these interventions will require a multifaceted approach, for which we have identified applicable intervention functions and policy categories, and have revealed preferred methods for intervention delivery.

These findings align with previous work assessing bowel care and quality of life following SCI [5–7, 30]. However, this is the first study to assess the barriers and facilitators to changing bowel care behaviour after SCI, and these are the first theory-based intervention recommendations to be co-developed for this issue. In addition, this study demonstrates the utility of the BCW and TDF for co-developing interventions into changing bowel care practices after SCI.

### Understanding the behaviour

The identification of ECR, BCon, social influences and knowledge as the most relevant TDF domains has clear implications for the future development of theory-based behaviour change interventions. Many behaviour change interventions solely address one’s motivation to change [15], and this approach fails to consider the environmental, social and knowledge-based factors that also influence behaviour. Our data suggest that individuals need personalised care that addresses access to knowledge and resources, while also addressing their beliefs about consequences and concerns around social influences, factors that may influence motivation to change bowel care practices. Future interventions should explore theories that leverage all COM-B components identified in this work to enable bowel care changes aimed at increasing satisfaction and quality of life.

### Environmental context and resources

Themes emerging from ECR explored the role of employment after SCI, access to support and resources and one’s opportunity or circumstance to change bowel care. As ECR relates to physical opportunity, this domain provides information on how one is physically able to interact with their environment and access available resources to change their bowel care [10]. Previous research assessing service needs after SCI showed that access to health care services was unmet within this population [31]. In addition, a lack of financial support, flexibility and time to change bowel care was a common theme within these interviews. This finding is unsurprising given the personal economic implications of SCI [32], the prevalence of fixed incomes in disabled communities [31] and the impact of fixed community care hours on care routines [33]. Interestingly, participants did not discuss the impact or limitation of care-aid hours and schedules as a barrier to changing bowel care. However, access to external in-home supports should be considered when developing interventions, as care-aid availability can be a limitation where changing bowel care requires additional time to allow for the adoption of a new routine.

These data also revealed the importance of workplace flexibility when considering changes to bowel care practices. Given that changes to employment often occur after SCI [34], it would be interesting to assess the interaction of care routines and workplace flexibility in future studies. Certainly, these findings highlight the need for increased flexibility in the workplace to allow individuals with SCI to attend to care needs.

One’s circumstance or opportunity to change bowel care was also a common theme within the ECR domain. A notable finding was the interplay between bladder and bowel care in the context of changing bowel care. Properly managed bladder function was shown to facilitate changes to bowel care, particularly in women. This discrepancy might reflect that the methods used for bladder drainage differ between men and women, with an increased need for transfer and intermittent catheterisation in women. It was also clear there were hierarchical considerations to changing care routines, with changes to bladder care taking priority over changes to bowel care, complementing previous research that showed that individuals with SCI will adopt fluid restriction to help with troublesome bladder care and urinary incontinence, even if it negatively impacts other aspects of quality of life, bladder health or bowel care routines [5]. This study highlights the importance of understanding care routines in context with each other, and underscores the need for holistic care.

### Beliefs about consequences

BCon addresses how one’s reflective motivation [10] or appraisal of whether there would be negative or positive consequences to changing bowel care influence the behaviour. Negative outcomes commonly discussed were the fear of bowel accidents and/or the uncertainty of the outcomes when changing care. Faecal incontinence is common and a key area of concern after SCI [5, 6, 35, 36]. Despite a variety of bowel care approaches, lesion levels, and injury severities, concerns about continence were common among all participants, as evident in our themes. Community partner feedback echoed this sentiment and highlighted the interplay between bowel care and self-esteem. Given the known impact of bowel care on quality of life after SCI [5–7, 30], it is perhaps not surprising that changing bowel care would also be perceived to have an impact on quality of life, and any perceived consequences profoundly impact changing bowel care.

### Social influences

It was not surprising these interviews discussed the unique power of peer mentorship as a prominent facilitator to changing bowel care practices because SCI peer mentorship has been shown previously to increase self-efficacy when assessing health care outcomes [28, 37]. Peer mentorship also plays an important role in increasing bowel care knowledge and facilitates adjustment after SCI [38, 39]. Peer mentorship has been described as providing increased credibility over that of non-peer relationships [40] providing a unique perspective that should be explored when developing interventions aimed at changing bowel care.

The influence of health care providers, family members and family support providers on making changes to bowel care was evident. These interpersonal dynamics have been increasingly shown to be a major influence on behaviour change after SCI [41–43]. Familial support plays an important role in rehabilitation and care, and relationship quality is related to levels of perceived social support [43]. In addition, having a live-in partner increases mobility and economic sufficiency, showing the multiple roles that family support has after SCI [39]. The perception of health care provider credibility also influenced bowel care behaviours. While perception or credibility differed between general practitioners and SCI specialists, access to specialist care was problematic, especially in remote, rural settings. This perception is an interesting consideration for the dissemination of bowel care strategies underscoring the need for health care providers to be informed about current best practices.

### Knowledge

Knowledge was discussed in relation to both knowledge of the physical changes to bowel and gastrointestinal function as a consequence of SCI and knowledge of the resources or supports available to aid bowel management (i.e., addressing how informed one is about options to change behaviour) [10]. As individuals with SCI commonly learn bowel care approaches during their in-patient rehabilitation, it is imperative that health care providers adhere to bowel care guidelines. Interestingly, clinical practice guidelines have poor adherence among health care professionals administering bowel care procedures unless they are targeted by a specific intervention [44]. Furthermore, adherence to clinical care guidelines is most beneficial when they reflect current best practices; unfortunately, this is not always the case, as there can be a significant lag between research discovery and adoption into clinical standards. For example, current bowel care guidelines incorporate advice concerning the use of local anaesthetics for the management of cardiovascular complications of bowel care [45], advice that directly contradicts recent data [46]. This finding highlights the need for evidence-based bowel management strategies and regular updates to clinical care guidelines. As knowledge creation is a key component to the knowledge-to-action framework of knowledge translation [47], it is important that knowledge tools are constantly evaluated so that their application can be appraised.

Interestingly, knowledge was identified as an important factor influencing the ability to make changes to bowel care, while the skills domain was not a prominent consideration in the interviews. This finding suggests that accessing information about bowel care change, not implementing changes, represents the greater barrier to change.

### Intervention and implementation options

There is an abundance of literature surrounding bowel management strategies and community-level assessments of bowel programmes that provides insight into how bowel care is currently being conducted in the community, with recent research advances showing promise for improving bowel care in controlled settings. However, the reality of implementing bowel care changes or advances into personal care routines is rarely studied. Given that bowel care is a key concern for individuals with SCI [5, 7, 48], it is imperative that changes and advances to care are also implemented and assessed in a community setting, shedding light on potential improvements to real-world bowel care after SCI. In addition, guidelines for at-home community neurogenic bowel management are dated and provide conflicting evidence [46, 49, 50]. Together these implementation gaps provide a unique opportunity to address prominent bowel care concerns.

### Future directions

We used the TDF to understand the barriers and facilitators underpinning behaviour change in the context of bowel care, enabling intervention recommendation, implementation and evaluation to be co-developed between researchers, community organisations and people with lived experience, with the ultimate aim of improving bowel care satisfaction. This work will require translation of these recommendations into concrete, actionable tools that can be evaluated using feasibility criteria [10]. It is important these future steps continue to use an integrated knowledge translation approach with community partnership to ensure relevant, informed and impactful decisions according to recently developed guidelines [18].

### Strengths and limitations

In addition to our integrated knowledge translation approach, this study used maximum variation sampling to aid the generalisability of our findings, but our scope was specific to individuals living with SCI in British Columbia, Canada. In spite of this limitation, we saw common themes emerge between participants regardless of lesion level, injury severity and geographical location suggesting that our findings are likely to be applicable to other individuals living with SCI in Canada and perhaps more broadly.

Our partnership with SCI BC, the co-development of the interview guide, and recommendations for intervention incorporated an integrated knowledge translation approach that increased the feasibility and relevancy of this work. In addition, this work employed the systematic use of validated behaviour change frameworks [9] that increases the efficacy and reproducibility of behaviour change interventions [15]. The results of both the deductive and inductive analyses were overseen by team members acting as critical friends in the research process and resonated with our community partners for whom they were identified as actionable and addressable concerns. These steps enhanced the rigour, sincerity, credibility and resonance of the inductive analysis [25].

Bowel care complications after SCI adversely impact the quality of life. Notably, the cardiovascular dysfunction that occurs concurrently with bowel care in people with high-level SCI has a profoundly negative impact on quality of life [5]. The nature of this study did not permit the investigation of cardiovascular autonomic dysfunction. However, in the future cardiovascular concerns should also be considered in the context of making changes to bowel care routines.

This work also revealed the unique impacts of caregivers and health care providers on changing bowel care practices, highlighting the need to examine the barriers and facilitators to supporting or recommending changes to bowel care in these individuals. Clearly, changing bowel care is multifaceted and future investigations need to address a myriad of concerns, including those experienced by health care professionals and caregivers.

Despite the use of maximum variation sampling, it is possible that our study was subject to selection bias. It was unclear whether participants chose to participate because they were experiencing profound bowel dysfunction at the time of the interview or were actively trying to change their bowel care. In addition, the proportion of women in this study exceeded that of the SCI demographic that may suggest that women have more continence concerns or more willingness to discuss continence concerns. Recruitment material suggested the interview could be conducted by someone with a female-gendered name that could have impacted willingness to participate.

## Conclusions

Bowel care is an area of dissatisfaction among individuals living with SCI. Numerous factors influence changing bowel care practices, especially those relating to the environment, resources, beliefs about consequences, social influences and knowledge. These data will enable the co-development of relevant and feasible theory-based bowel care interventions that support people with SCI to make changes to a modifiable behaviour and improve their quality of life.

## Supplementary information

Supplementary Table 1

## Data Availability

Due to legal and ethical restrictions, data cannot be made publicly available. Data will be made available upon request; however, only aggregated data may be in the public domain according to the stipulations from our research ethics board with respect to the maintenance of confidentiality. Additional published or public analyses would only be permitted with ethics approval for secondary data access, and only with aggregated analyses. Requests can be sent to Jeff Toward, Director, Office of Research Ethics, Simon Fraser University (jtoward@sfu.ca).
