# Supplementary material for: Barriers and facilitators to changing bowel care practices after spinal cord injury: a Theoretical Domains Framework approach
Source: Spinal Cord. 2022 Jan 7;60(7):664–73. doi: 10.1038/s41393-021-00743-0 (PMC9287175; doi:10.1038/s41393-021-00743-0)
Supplement: Supplementary file 1 — Supplementary Table 1 [file 41393_2021_743_MOESM1_ESM.docx]

**Supplemental Table 1 – *Integrated Knowledge Translation Process.***

| **Research Stage** | **Type of Activity** | **Activity Description** |
| --- | --- | --- |
| **Identification of knowledge partners** | Discussion  (In person) | Consistent with the guiding principles of integrated knowledge translation and based on the geographical location of the study, Spinal Cord Injury British Columbia (SCI BC) (CMB) and local SCI clinicians (RW, MM) were identified as research users and engaged as partners throughout the research process.  **Partner description** For more than 60 years, SCI BC has been helping individuals with SCI living in British Columbia to adapt, adjust and thrive, and is British Columbia’s preeminent organization for peer support and information about living well with an SCI. SCI BC has a provincial membership of >2,500 individuals and has a long-standing history of partnering with academic researchers. They regularly communicate with more than 4,000 people, including individuals with SCI, family members and caregivers, health professionals, and other community and provincial stakeholders. |
| **Conceptual Design** | Discussion (In person and video calls) | The research question and methodology were developed in partnership with the broader team including VL, VC, HG, RW, MM, and CMB prior to writing a funding application. |
| **Conceptual Design** | Discussion (Video call) | Once funding was received, VL, VC, HG, CMB, and RM finalized methodology decisions including recruitment goals, interview instructions and data analysis |
| **Recruitment** | Developing & distributing recruitment materials (Email) | VL created templates for recruitment posters, social media recruitment, and consent forms. All materials were reviewed and edited by VC.  HG, and CMB reviewed, edited and approved recruitment materials and procedures. Once approved, CMB distributed recruitment materials. |
| **Data Collection** | Conducting interviews (Phone call) | VL scheduled and recorded all 13 interviews. |
| **Data Analysis, Interpretation, & Dissemination** | Discussion  (Video call) | VL and RM presented the developed coding to VC and HG, who both made recommendations to refine the coding approach. VL conducted the inductive analysis and refined themes with RM, HG, and VC.  VL presented the results of deductive and inductive coding to CMB, RW, and MM where all discussed the interpretation of the results.  VL presented the findings to SCI BC (with VC and CMB also present) and discussed the resonance of findings with SCI BC members external to the research team. VL prepared the presentation of findings that was reviewed by VC. |
| **Manuscript Preparation** | E-mail | All co-authors reviewed the manuscript, provided feedback, and approved final content prior to submission for publication. |
